# Mouse CD11b^+^Kupffer Cells Recruited from Bone Marrow Accelerate Liver Regeneration after Partial Hepatectomy

**DOI:** 10.1371/journal.pone.0136774

**Published:** 2015-09-02

**Authors:** Kiyoshi Nishiyama, Hiroyuki Nakashima, Masami Ikarashi, Manabu Kinoshita, Masahiro Nakashima, Suefumi Aosasa, Shuhji Seki, Junji Yamamoto

**Affiliations:** 1 Department of Surgery, National Defense Medical College, Namiki 3–2, Tokorozawa, Saitama, Japan 359–8513; 2 Department of Immunology and Microbiology, National Defense Medical College, Namiki 3–2, Tokorozawa, Saitama, Japan 359–8513; Montana State University, UNITED STATES

## Abstract

TNF and Fas/FasL are vital components, not only in hepatocyte injury, but are also required for hepatocyte regeneration. Liver F4/80^+^Kupffer cells are classified into two subsets; resident radio-resistant CD68^+^cells with phagocytic and bactericidal activity, and recruited radio-sensitive CD11b^+^cells with cytokine-producing capacity. The aim of this study was to investigate the role of these Kupffer cells in the liver regeneration after partial hepatectomy (PHx) in mice. The proportion of Kupffer cell subsets in the remnant liver was examined in C57BL/6 mice by flow cytometry after PHx. To examine the role of CD11b^+^Kupffer cells/Mφ, mice were depleted of these cells before PHx by non-lethal 5 Gy irradiation with or without bone marrow transplantation (BMT) or the injection of a CCR2 (MCP-1 receptor) antagonist, and liver regeneration was evaluated. Although the proportion of CD68^+^Kupffer cells did not significantly change after PHx, the proportion of CD11b^+^Kupffer cells/Mφ and their FasL expression was greatly increased at three days after PHx, when the hepatocytes vigorously proliferate. Serum TNF and MCP-1 levels peaked one day after PHx. Irradiation eliminated the CD11b^+^Kupffer cells/Mφ for approximately two weeks in the liver, while CD68^+^Kupffer cells, NK cells and NKT cells remained, and hepatocyte regeneration was retarded. However, BMT partially restored CD11b^+^Kupffer cells/Mφ and recovered the liver regeneration. Furthermore, CCR2 antagonist treatment decreased the CD11b^+^Kupffer cells/Mφ and significantly inhibited liver regeneration. The CD11b^+^Kupffer cells/Mφ recruited from bone marrow by the MCP-1 produced by CD68^+^Kupffer cells play a pivotal role in liver regeneration via the TNF/FasL/Fas pathway after PHx.

## Introduction

The liver has long been known to have high regenerative activity, and since the 70% partial hepatectomy (PHx) experiment was reported by Higgins and Anderson in 1931 [1], many studies have been performed regarding the mechanisms of liver regeneration [2–5]. However, the role of liver Kupffer cells in liver regeneration after PHx remains to be elucidated.

It is known that hepatocyte proliferation in mice starts around 32 h, and peaks around two to three days after PHx, and liver regeneration ends up to 10 days after PHx. At the end of liver regeneration, the liver weight recovers to that before PHx [4, 6, 7], although the shape of liver after PHx is different from that before PHx. Although hepatocytes themselves obviously have regenerative activity, it is now generally considered that paracrine factors, such as liver leukocytes, sinusoidal endothelial cells, cytokines (IL-6, TNF) and chemokines may also be involved in the liver regeneration [7–12]. Hepatocyte proliferation after PHx was found to be significantly inhibited in TNF-deficient mice, TNF-receptor-deficient mice and Fas or FasL-deficient/depleted mice, thus suggesting that these molecules are involved in liver regeneration [7, 13–15].

We previously reported that NKT cells activated by their synthetic ligand (alpha-galactosylceramide, α-GalCer) [16, 17] express FasL induced by TNF, and evoke a severe injury of hepatocytes expressing Fas, especially in aged mice, in which TNF is produced by CD11b^+^ Kupffer cells/macrophages (Mφ) (TNF/FasL/Fas pathway) [18–20]. In sharp contrast, liver NKT cells express FasL, which accelerates the hepatocyte proliferation after PHx via the same TNF/FasL/Fas pathway, especially when NKT cells are activated by α-GalCer [7]. These findings suggest that NKT cells expressing FasL may induce apoptosis in old or damaged hepatocytes while increasing the proliferation of newly generating hepatocytes to maintain the turnover of hepatocytes and homeostasis of the liver [7]. However, NK cells may inhibit hepatocyte proliferation after PHx, and may be involved in the termination of liver regeneration [7, 21].

We found that the liver F4/80^+^ Kupffer cells/Mφ in mice can be classified into two functionally and developmentally different subsets; one is a radio-resistant CD68^+^ subset with ROS-producing and bactericidal activities (resident CD68^+^ Kupffer cells) and the other is a radio-sensitive CD11b^+^ subset with cytokine- (IL-12, TNF) producing capacity, which is involved in antitumor immunity by producing IL-12 and in inflammation by producing TNF (recruited CD11b^+^ Kupffer/Mφ) [22, 23]. This subclassification is also essentially applicable to human liver Kupffer cells [23]. In addition, we have recently reported that CD11b^+^ Kupffer/Mφ are hepatotoxic effectors in carbon-tetrachloride (CCl_4_)-induced acute chemical hepatitis, in which CD11b^+^ Kupffer/Mφ produce both TNF and FasL and induce apoptosis/necrosis of chemically damaged hepatocytes, which was independent of NK cells and NKT cells [24]. Based on these findings of liver immune cells and Kupffer cells/Mφ, we hypothesized that, similar to the case of NKT cells [7], CD11b^+^ Kupffer/Mφ may also be involved in liver regeneration using TNF/FasL. TNF has been considered to play a crucial role in liver regeneration, because abrogation of TNF by neutralizing antibody, or specific genetic down-regulation inhibits hepatic regeneration after PHx [25–27]. Although Kupffer cells were considered to be the most possible candidate of TNF producing cells, Kupffer cell depletion by gadolinium chloride (GdCl_3_) or liposome encapsulated clodronate up-regulated the TNF synthesis and accelerated the hepatic regeneration [28–30]. Based on these findings the source of TNF after PHx has been controversial for a long time [31]. The current study was designed to explore the producers of TNF and the role of TNF in the liver regeneration after PHx.

## Materials and Methods

All animal treatment including surgical procedure, whole body irradiation, subcutaneous implantation of osmotic pump, were approved by The Ethics Committee of Animal Care and Experimentation, National Defense Medical College, Japan (Permission number: 15001).

### Mice

The eight-week-old C57BL/6 mice were purchased from CLEA-Japan, Inc. (Tokyo Japan) and were subjected to experiments until the age of ten weeks. Throughout the experiments, mice were maintained in temperature and humidity controlled specific pathogen free environment. Sterilized chow and water were accessed freely. For the experimental design, they were randomly divided into each experimental group in which 3 to 6 mice were included.

### Partial hepatectomy

The mice underwent 70% partial hepatectomy (PHx) by a resection of the anterior and left lateral lobes of the liver under deep inhalation anesthesia using Isoflurane according to the method described by Higgins and Anderson [32]. Before surgical closure of the peritoneal space, about 300 μL of phosphate-buffered saline (PBS) or antibody aliquots were administered into the peritoneal space. Control mice were subjected to a sham operation and received PBS.

### Isolation of mononuclear cells (MNC)

The mice were sacrificed under deep Isoflurane anesthesia at the indicated times after PHx, and the remnant liver was removed. Hepatic MNC were prepared essentially as described in a previous report without collagenase treatment[33]. In some experiments, to harvest the liver MNC, including CD68^+^ Kupffer cells, livers were minced and suspended in HBSS containing 0.05% collagenase (Wako, Osaka, Japan), and then were shaken for 20 min in a 37°C water bath. Next, the liver specimens were washed in 1% FBS RPMI 1640 and then filtered through a stainless steel mesh. After being mixed in isotonic 33% Percoll solution containing heparin, the samples were centrifuged for 15 min at 500 × g at room temperature. After removing the supernatant, the pellets were resuspended in a red blood cell lysis solution and then were washed twice in 1% FBS RPMI 1640 [22].

### Flow cytometric analysis

Before staining with antibodies, the murine liver MNC were incubated with Fc-blocker (2.4 G2; BD PharMingen, San Diego, CA) to prevent any nonspecific binding. The MNC were stained using a FITC-conjugated anti-F4/80 Ab (BM8; eBioscience, San Diego, CA), PECy5-conjugated anti-CD11b Ab (M1/70; eBioscience) or Biotin-conjugated anti-CD68 Ab (FA-11: AbD Serotec, Oxford, UK) with PE-streptavidin. Flow cytometric analysis using an FC500 instrument (Beckman Coulter, Miami, Fl).

### Measurement of the serum cytokines and a chemokine, MCP-1 (monocyte chemotactic protein-1)

The serum TNF, IL-12, IFN-γ and MCP-1 (CCR2 ligand) levels were measured by ELISA (BD Bioscience, San Diego, CA). Blood samples were obtained from the inferior vena cava when mice were sacrificed.

### Intracellular staining for TNF

MNC were incubated with BD GolgiStop (0.7 μg/ml, BD PharMingen) for four hours before staining. After incubation with Fc-blocker, the cells were stained with a FITC-conjugated anti-F4/80 Ab and PECy5-conjugated anti-CD11b Ab, or a biotin-conjugated anti-CD68 Ab with PECy5-streptavidin. Subsequently, the cells were incubated with BD Cytofix/Cytoperm solution (BD Pharmingen) at 4°C for 20 min, and then were washed with BD Perm/Wash solution (BD Pharmingen). Thereafter, the cells were stained with a PE-conjugated anti-TNF mAb (eBioscience) or isotype rat IgG1 Ab (eBioscience) at 4°C for 20 min, and then were analyzed using the FC500 instrument.

### Whole body X-ray irradiation and bone marrow transplantation

The mice were exposed to 5 Gy whole body irradiation, which was given at a dose rate of 0.4 Gy/min at 150 kV and 5 mA (HITACHI MBR-1505R2, Tokyo, Japan). Some of these irradiated mice received 1×10^7^ femoral and tibial bone marrow cells immediately after irradiation, and PHx was performed five days after irradiation, when the CD11b^+^ Kupffer cells/Mφ were mostly eliminated.

### Continuous injection of a CCR2 antagonist into mice

The CCR2 antagonist (Cas 445479-0-Calbiochem) was purchased from Merc Milllipore, Merck KGaA (Darmstadt, Germany). A total of 560 μg of CCR2 antagonist in 200 μl of DMSO was continuously injected for seven days using a MINI-OSMOTIC PUMP MODEL 2001 (DURECT Corporation, Cupertino, CA), which was implanted subcutaneously in the back of mice three hours before PHx.

### Histological assessment

The collected liver specimens were fixed in 10% formalin and were stored at 4°C and embedded in paraffin. Sections were stained with hematoxylin-eosin for histological examination. Total number of hepatocytes and mitotic figures were counted in a same field of 400× magnification. After counting in randomly selected 20 fields, mitosis figures per 2000 hepatocytes were calculated.

### Statistical analysis

All quantitative data in this study were expressed as the means ± SE from at least 3 independent experiments. Where appropriate, Student’s *t*-test was employed to compare the data for two different groups. Values of *P* < .05 were considered to be significant.

## Results

### The increase of CD11b^+^ Kupffer cells/Mφ during liver regeneration after PHx

**Fig 1:**
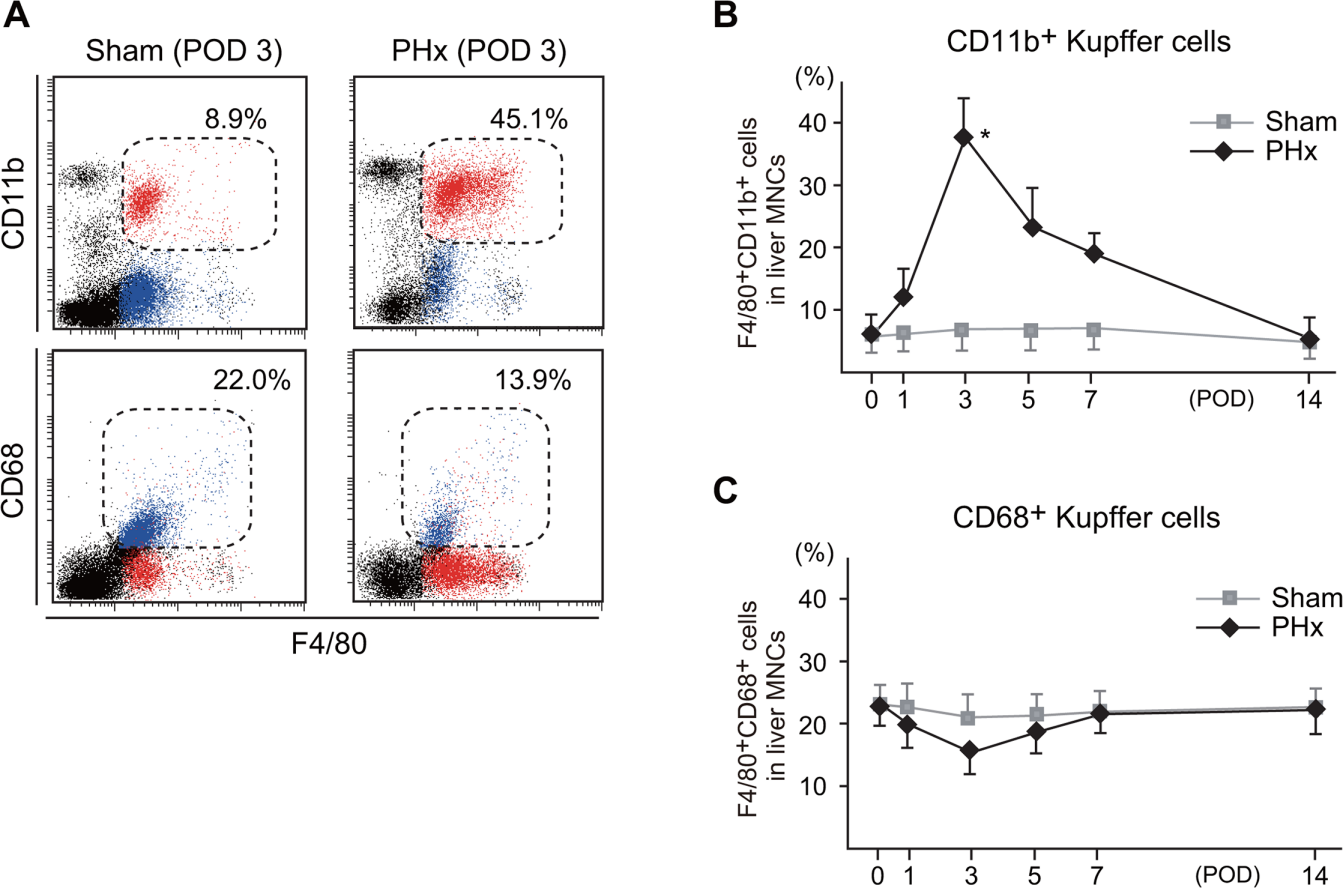
The flow cytometric analysis of liver F4/80^+^CD11b^+^ cells and F4/80^+^CD68^+^ cells three days after Sham operated or PHx mice **(A). The changes in the proportions of F4/80** ^**+**^ **CD11b** ^**+**^ **cells in the remnant livers (B). The changes in the proportions of F4/80** ^**+**^ **CD68** ^**+**^ **cells in the remnant livers (C).** The percentages of F4/80^+^CD11b^+^ cells which are indicated by red dots and F4/80^+^CD68^+^ cells indicated by blue dots are the representative data from four to six mice (A). The percentages of each cellular population at the indicated time points are shown as the means±SE (B, C). (*P < .05 vs Sham).

The proportion of F4/80^+^CD11b^+^ Kupffer cells/Mφ in liver MNCs increased and peaked at three days after PHx (approximately 45% of liver MNC) and thereafter gradually decreased (Fig 1A and 1B). The CD68^+^ Kupffer cells was relatively decreased at three days after PHx presumably by the increase of CD11b^+^ Kupffer cells but there was not the statistical difference (Fig 1C). These findings were not observed in the Sham operated mice. All mice were survived after surgical operation and had no sign of serious adverse side effect.

### The serum chemokine and cytokine levels during liver regeneration after PHx

**Fig 2:**
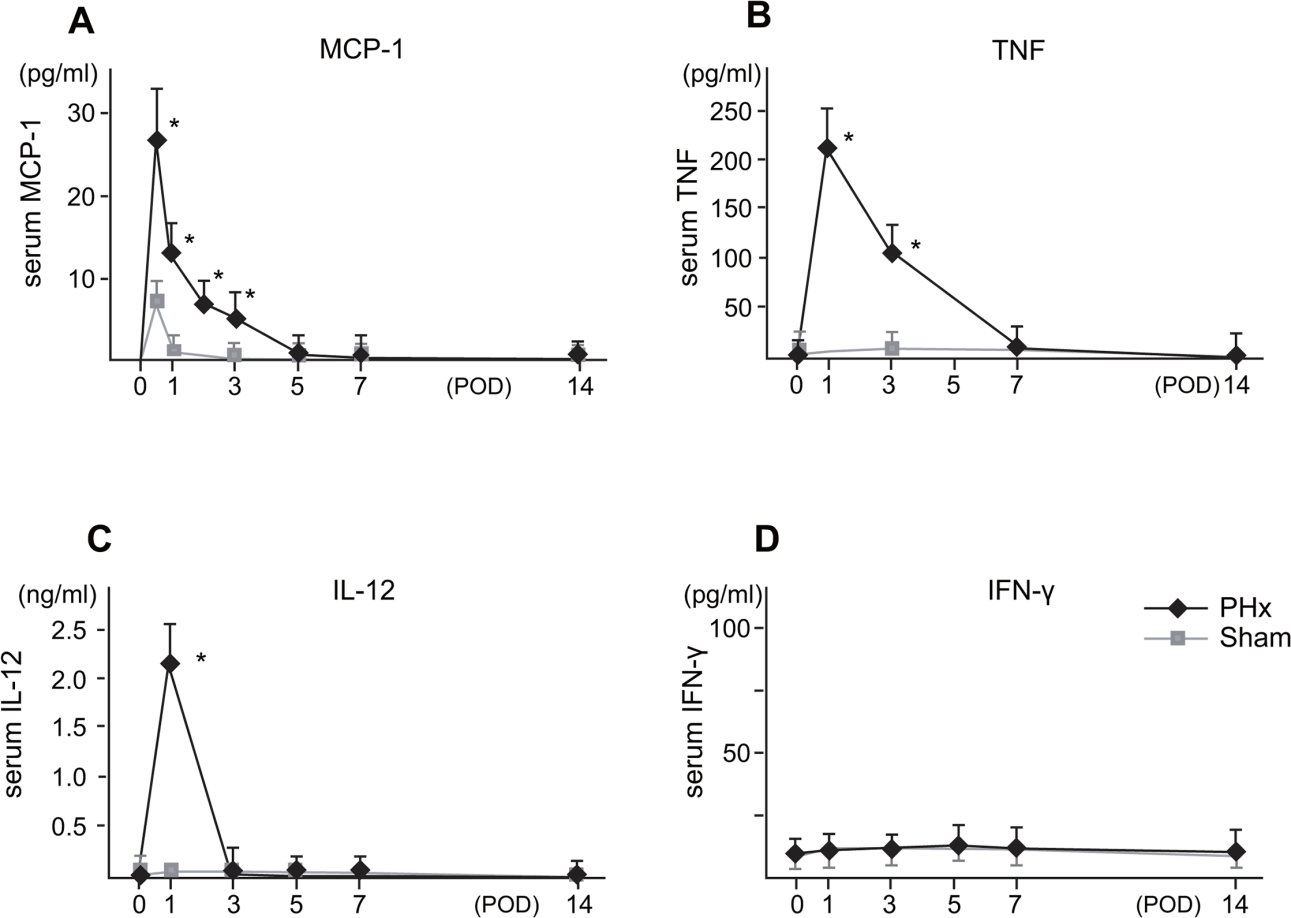
The serum cytokine levels and MCP-1 levels after PHx (A-D). The cytokine and MCP-1 levels of sera of three to five mice were examined at each of the indicated time points. The data are shown as the means±SE from fout to six mice. (*P < .05 vs Sham).

The levels of serum MCP-1 presumably produced by CD68^+^ Kupffer cells [23, 24] increased and peaked at 12 h after PHx and decreased thereafter (Fig 2A), suggesting that MCP-1 binds to CCR2 on CD11b^+^ Kupffer cells/Mφ and leads to their accumulation in the liver from the periphery and bone marrow [34].The levels of serum TNF and IL-12 presumably produced by CD11b^+^ Kupffer cells/Mφ were increased one day after PHx, while the serum IFN-γ levels did not show a significant change (Fig 2B–2D).

### The increased intracellular TNF production and FasL expression in CD11b^+^ Kupffer cells/Mφ during liver regeneration after PHx

**Fig 3:**
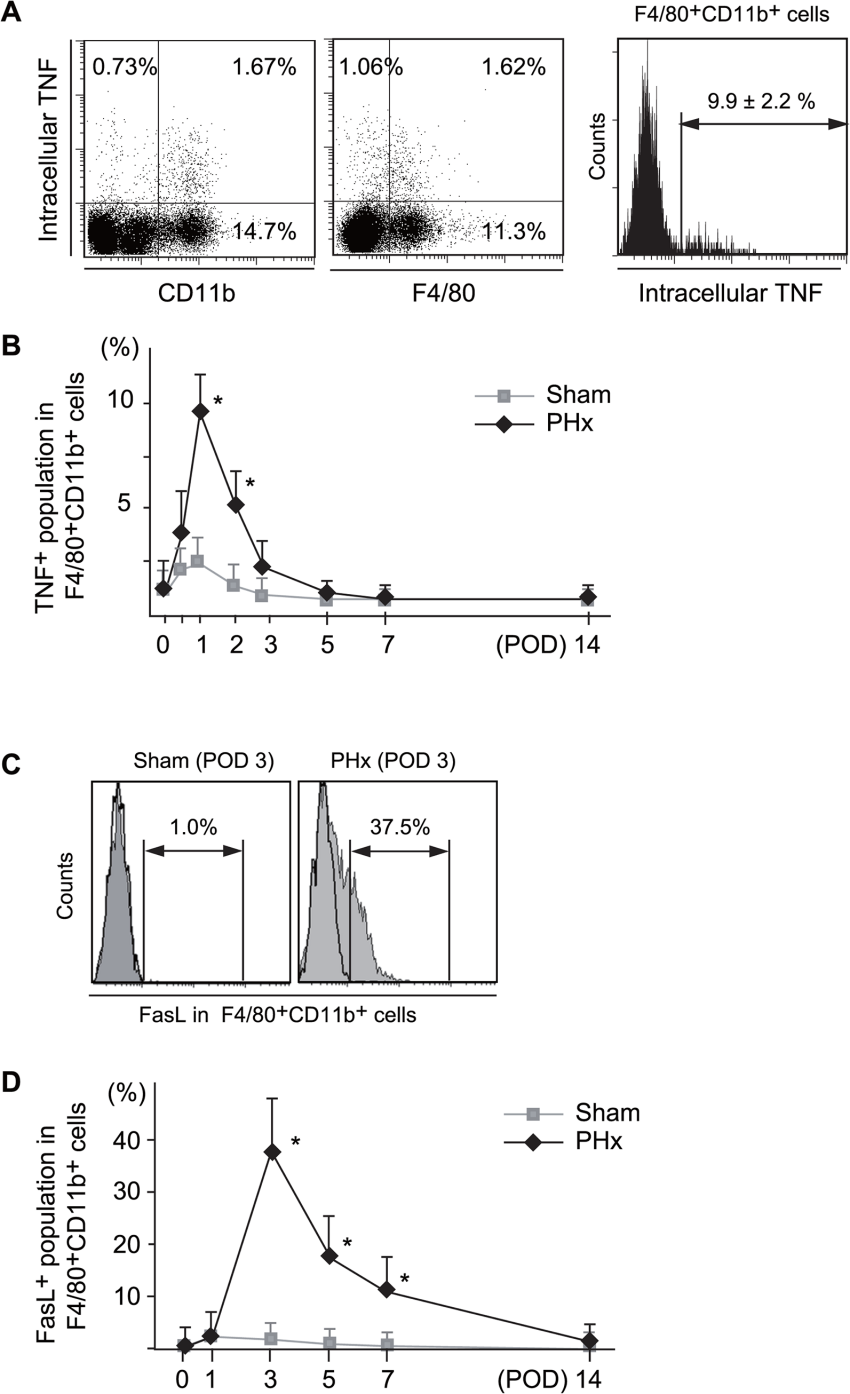
The flow cytometric analysis of the intracellular TNF-producing (A, B) and surface FasL^+^ expression (C, D) of CD11b^+^ Kupffer cells after Sham operated or PHx mice. **The changes in the proportions of intracellular TNF (B) and FasL (D) positive CD11b** ^**+**^ **Kupffer cells in the remnant livers.** The dot plot analysis of intracellular TNF and CD11b or F4/80. F4/80^+^CD11b^+^ cells are gated and intracellular TNF was examined by histogram plot (A, right panel). Surface FasL was examined in gated F4/80^+^CD11b^+^ cells in remnant liver and the representative data from three to five mice were shown (C). The percentages of intracellular TNF or surface FasL positive cells in F4/80^+^CD11b^+^ cells at the indicated time points are shown as the means±SE from three to five mice (B, D). (*P < .05 vs Sham).

The intracellular TNF production of CD11b^+^ Kupffer cells/Mφ increased immediately after PHx and peaked at 24 h after PHx (9.9%) (Fig 3A and 3B). Dot blot analysis showed that F4/80 and CD11b positive population is the major population of TNF producing cells. Similarly, the FasL expression of CD11b^+^ Kupffer cells/Mφ proportionally peaked at three days after PHx (37.5%) (Fig 3C and 3D). These findings were not observed in the Sham operated mice (S1 Fig).

### The effect of irradiation with or without bone marrow transplantation (BMT) on the liver regeneration

**Fig 4:**
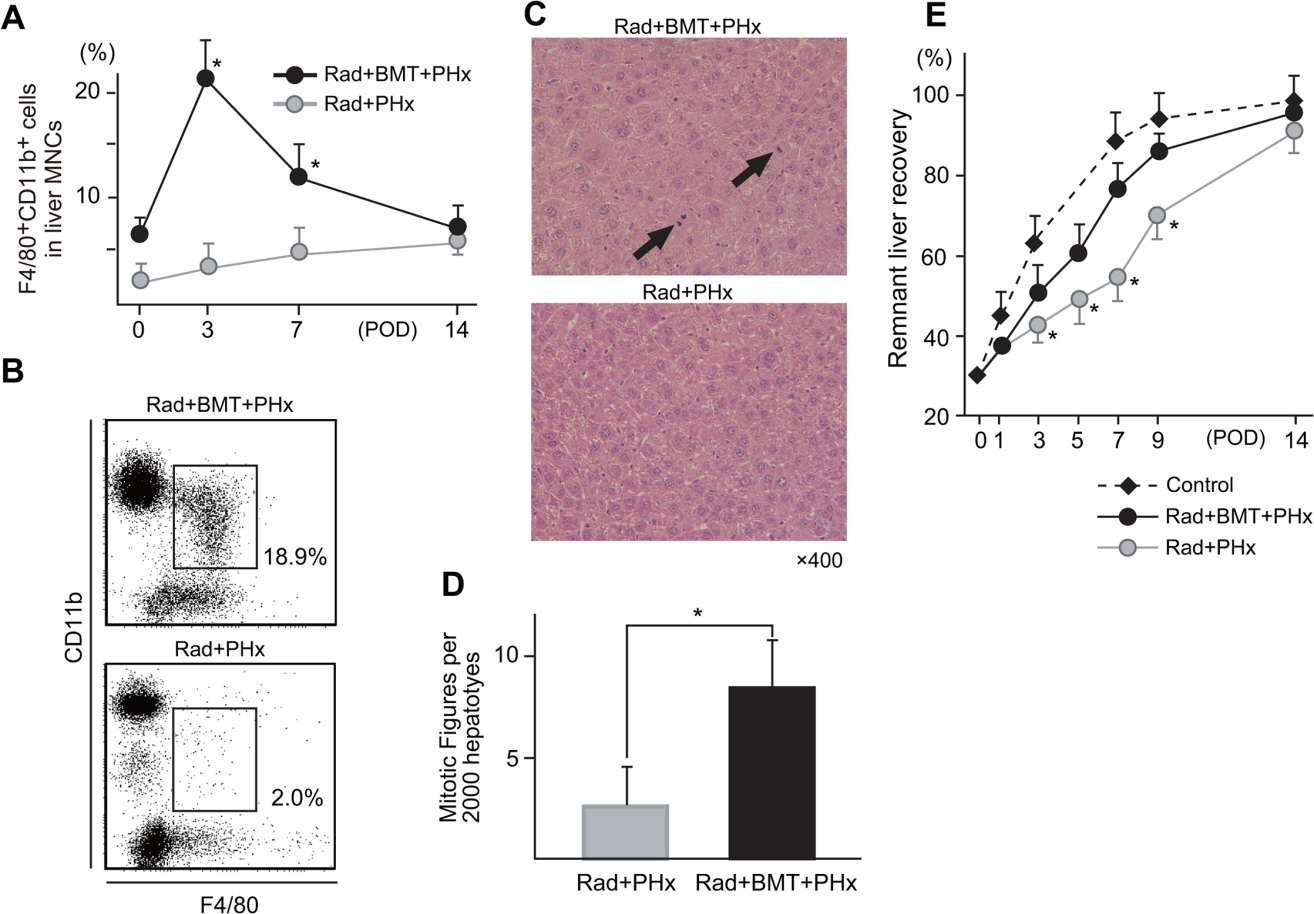
The changes in the proportions of CD11b Kupffer cells in 5 Gy-irradiated mice with or without BMT **(A). Representative flow cytometric data for the CD11b Kupffer cells three days after PHx with or without BMT (B). The mitotic figures of hepatocytes (C, D). The remnant liver weight recovery in irradiated mice with or without BMT after PHx (E).** Three to five mice were examined at each of the indicated time points. (*P < .05 vs Rad+BMT+PHx). Total number of hepatocytes and mitotic figures were counted in a same field of 400× magnification. After counting in randomly selected 20 fields, mitosis figures per 2000 hepatocytes were calculated.

Although 5 Gy radiation is not lethal and almost all mice can survive the irradiation, it can deplete the CD11b^+^ Kupffer cells/Mφ from five days up to 12 days after irradiation, because the CD11b^+^ Kupffer cells/Mφ are radio-sensitive, while CD68^+^ Kupffer cells are radio-resistant [23]. Similarly, after PHx in irradiated mice, the CD11b^+^ Kupffer cells/Mφ were selectively depleted (Fig 4A and 4B), while CD68^+^ Kupffer cells proportionally increased (S2 Fig). NK cells and NKT cells, especially NKT cells, are also radio-resistant (S3 Fig). Performing BMT immediately after irradiation led to recovery of the CD11b^+^ Kupffer cells/Mφ (eight days after irradiation and three days after PHx) (Fig 4A and 4B). In addition, the mitotic figures of hepatocytes were decreased by irradiation, whereas BMT led to a recovery of the hepatocyte mitotic figures (Fig 4C and 4D). Consistently, the livers of irradiated mice showed impaired and retarded regeneration, while bone marrow transplantation significantly recovered the liver regeneration (Fig 4E). All mice were survived after irradiation and had no sign of serious adverse side effect in each group.

### The effects of continuous CCR2 antagonist infusion by an osmotic pressure pump on the liver regeneration after PHx

**Fig 5:**
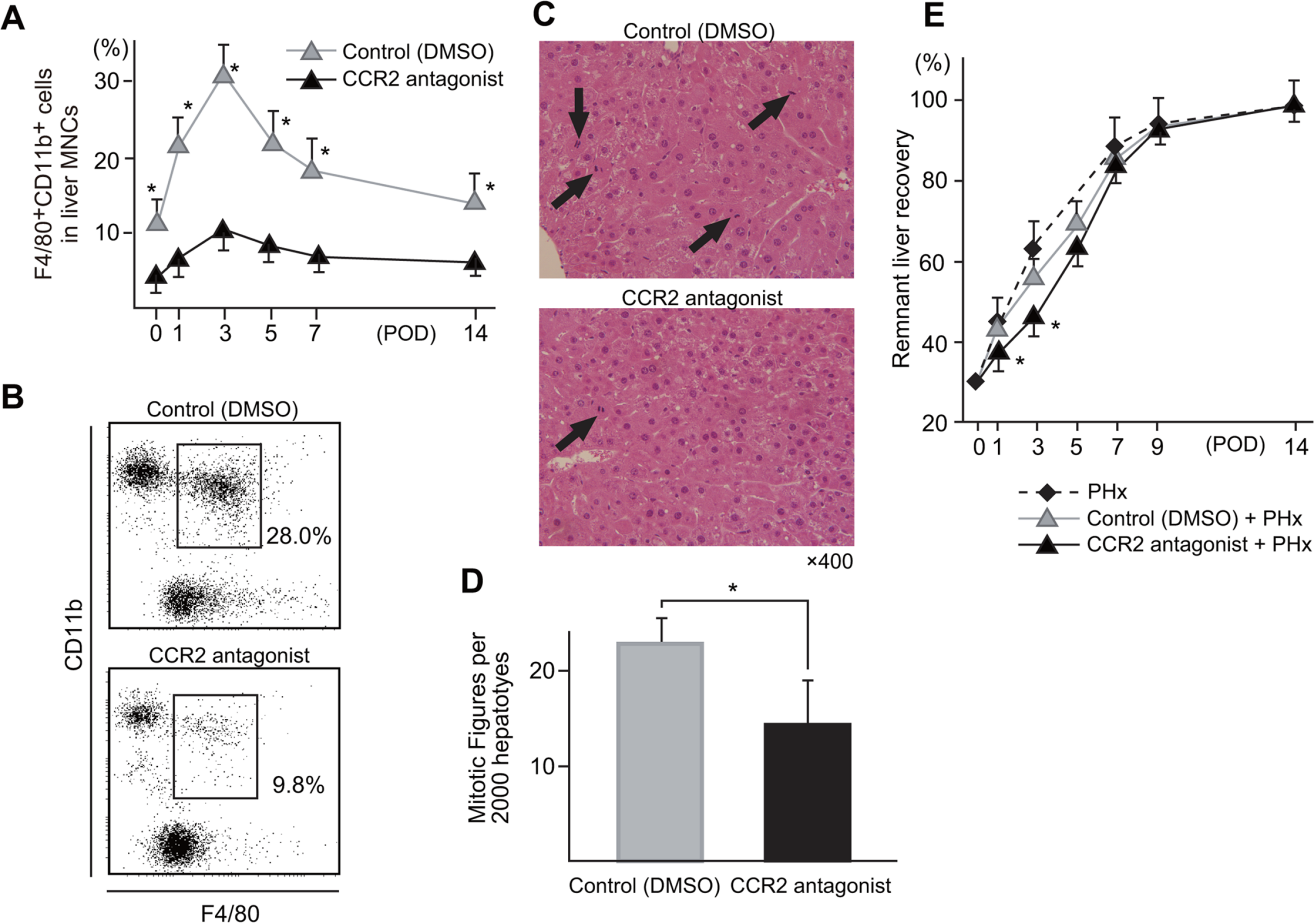
The changes in the proportions of CD11b Kupffer cells after PHx in mice with or without CCR2 antagonist administration **(A). Representative flow cytometric data for CD11b Kupffer cells three days after PHx with or without treatment with a CCR2 antagonist (B). The mitotic figures of hepatocytes (C, D). The remnant liver weight recovery in mice with or without CCR2 antagonist treatment after PHx (E).** Three to five mice were examined at each of the indicated time points. (*P < .05 vs control).

To further confirm the role of CD11b^+^ Kupffer cells/Mφ, we examined the effects of an antagonist of CCR2 [35, 36], which is a ligand of MCP-1 produced by CD68^+^ Kupffer cells that leads to the accumulation of CD11b^+^ Kupffer cells/Mφ into the liver from the periphery and BM [23, 24]. The results showed that the continuous administration of CCR2 antagonist greatly decreased the accumulation of CD11b^+^ Kupffer cells/Mφ (Fig 5A and 5B) and also decreased the hepatocyte proliferation after PHx (Fig 5C and 5D). Consequently, the liver regeneration was significantly impaired by CCR2 antagonist treatment, especially early after PHx (at one and three days post-PHx) (Fig 5E).

### The time course of the changes in the proportions of NK cells and NKT cells during liver regeneration

**Fig 6:**
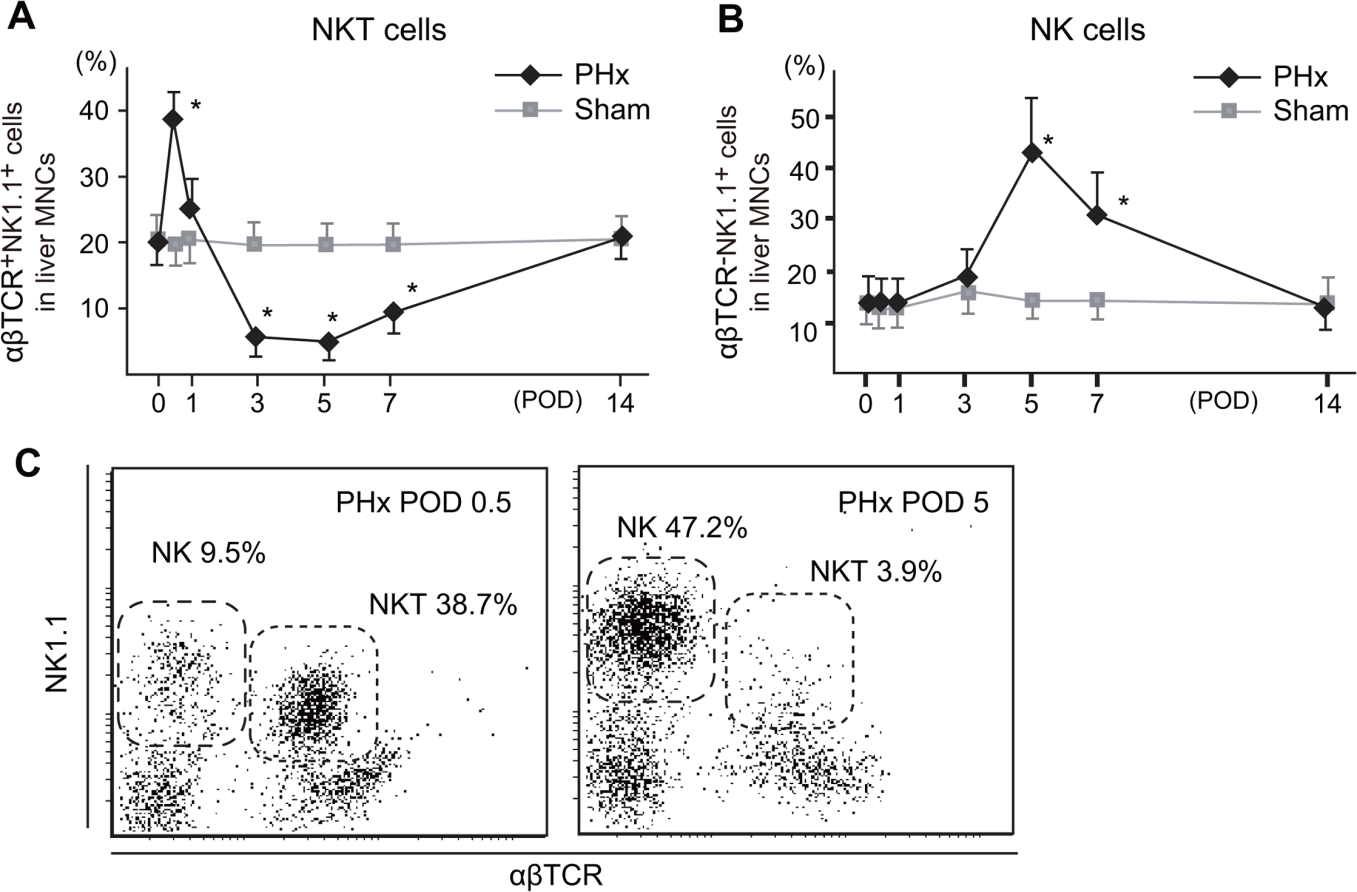
The changes in the proportions of NKT cells and NK cells in the remnant livers after PHx (A, B), and a flow cytometric analysis of NKT and NK cells (C). The percentages of NKT cells and NK cells from three to five mice at each of the indicated time points are shown as the means±SE (A, B), and representative data are shown four experiments with similar results (C). (*P < .05 vs Sham).

It was previously reported that NKT cells expand in the early phase (12h) after PHx [37] and we previously reported that ligand-activated NKT cells accelerate liver regeneration after PHx [7], we also examined whether NKT cells increased and may participate in liver regeneration after PHx. The results denonstrated that NKT cells indeed proportionally increased immediately after PHx (12h after PHx) to comprise up to 40% of liver MNC, and decreased thereafter (Fig 6A and 6C). On the other hand, the proportion of NK cells gradually increased and peaked at five days after PHx (up to 50%), and thereafter gradually decreased (Fig 6B and 6C).

### Increased antitumor cytotoxicity of liver MNC at seven days after PHx

The liver MNC at seven days after PHx showed greatly increased antitumor cytotoxicity against Yac-1 and EL-4 tumor cells (S4 Fig). When PHx mice (at seven days after PHx) were i.v. injected with EL4 liver metastatic tumors, PHx mice showed increased survival rate as compared to that of sham mice, which was not observed when mice were injected with tumors at three days after PHx (S5 Fig). These results suggest that liver NK cells at seven days after PHx are functionally activated.

## Discussion

In the present study, we demonstrated that the CD11b^+^ Kupffer cells/Mφ recruited from the periphery and bone marrow were greatly increased in the liver after PHx, and these produced TNF and FasL and accelerated liver regeneration. This was consistent with our previous observation that the neutralization of both TNF and FasL inhibited liver regeneration after PHx [7].

The mouse liver contains several innate immune mononuclear cells, which include NK cells (10–15%), NKT cells (15–20%), B cells (30–40%) and Kupffer cells (approximately 20%) [18, 38–40]. The human liver also contains NK cells (20–30%), NKT cells (CD56+T cells) (20%), T cells, B cells and Kupffer cells [23, 41]. Liver NK cells and NKT cells play an important role in the antitumor immunity induced by IL-12, α-GalCer [18, 19, 38, 42] or bacterial DNA (CpG-ODN)[43]. These cells also play a pivotal role in anti-bacterial immunity by producing IFN-γ [33, 43]

In addition, we have recently explored the detailed characteristics of CD68^+^ Kupffer cells and CD11b^+^ Kupffer cells/Mφ. CD68^+^ Kupffer cells are radio-resistant cells derived from c-kit^+^CD32^+^ precursor cells in the liver, and have a potent phagocytic and bactericidal activity, while CD11b^+^ Kupffer cells/Mφ are radio-sensitive cells derived from bone marrow that have a potent cytokine (IL-12, TNF)-producing capacity and are profoundly involved in the antitumor immunity exerted by NK cells and NKT cells [22, 23] by producing IL-12. Further, CD68^+^ Kupffer cells are spindle-shaped and larger than CD11b^+^ Kupffer cells/Mφ, and cannot be harvested without collagenase treatment of liver specimens. In contrast, CD11b^+^ Kupffer cells/Mφ are small and round-shaped and can be easily obtained without collagenase treatment of the liver [23]. Therefore, CD68^+^ Kupffer cells and CD11b^+^ Kupffer cells/Mφ are functionally and developmentally quite different.

Liver immune cells have also been shown to play a pivotal role in several experimental hepatitis models. α-GalCer and CpG-ODN not only induce antitumor immunity in the liver by activating NK cells via IL-12 (produced by CD11b^+^ Kupffer cells/Mφ), but also induce severe hepatic injury by NKT cells, especially in aged mice and mice fed a high-fat and high cholesterol diet, which occurred via the FasL/Fas pathway stimulated by TNF (produced by CD11b^+^ Kupffer cells/Mφ) [19, 20, 42, 43]. In the case of Concanavalin-A-induced hepatitis, the ROS produced by CD68^+^ Kupffer cells is the final effector, while the TNF produced by CD11b^+^ Kupffer cells/Mφ and NKT cells is needed to activate and increase the CD68^+^ Kupffer cells [44]. Interestingly, the NKT cells activated by α-GalCer in turn accelerate the proliferation of newly regenerating hepatocytes after PHx [7].

Although Kupffer cells have been suggested to be involved in the hepatic injury induced by a hepatotoxic chemical, carbon tetrachloride (CCl_4_) [34, 45–48], much remained to be elucidated with regard to the functions of these cells. We have recently found that the TNF and FasL produced by liver CD11b^+^ Kupffer cells/Mφ are final effectors in CCl_4_-induced hepatic injury, while neither NKT cells nor NK cells are involved in this hepatitis [24]. In addition, unexpectedly, the depletion of CD68^+^ Kupffer cells by clodronate liposome pretreatment increased the CD11b^+^ Kupffer cells/Mφ cell population and aggravated CCl_4_-induced hepatic injury, because the MCP-1 (CCL2) produced by CD68^+^ Kupffer cells (before they underwent apoptosis following clodronate liposome phagocytosis) increased the accumulation and activation of CD11b^+^ Kupffer cells/Mφ [24, 49].

Since NKT cells can be either hepatotoxic effectors or hepatocyte growth effectors depending on the conditions of the liver [18–20, 42, 44], we also examined the role of CD11b^+^ Kupffer cells/Mφ in the liver regeneration after PHx. Surprisingly, but not unexpectedly, the CD11b^+^ Kupffer cells/Mφ greatly increased three days after PHx (45% in liver MNC) and these produced TNF and expressed FasL, which accelerated hepatocyte proliferation, because a depletion or decrease of CD11b^+^ Kupffer cells/Mφ either by irradiation or a CCR2 antagonist significantly inhibited the liver regeneration. Several studies, including our previous study, showed that TNF, FasL and NKT cells are important paracrine factors involved in the hepatocyte proliferation after PHx [7, 13, 15, 32].

In addition, the MCP-1 produced by CD68^+^ Kupffer cells [23, 24] early after PHx interacts with CCR2 on monocytes from the blood and bone marrow, and leads to their accumulation into the liver as CD11b^+^ Kupffer cells/Mφ [50]. However, as described above, the depletion of CD68^+^ Kupffer cells by clodronate liposome or GdCl_3_ increased the CD11b^+^ Kupffer cells/Mφ, because CD68^+^ Kupffer cells produce MCP-1 before undergoing apoptosis, and MCP-1 induces the accumulation of CD11b^+^ Kupffer cells/Mφ into the liver [23, 24]. Furthermore, we previously reported that the depletion of CD68^+^ Kupffer cells by GdCl_3_ augmented the hepatocyte proliferation (mitotic figures) after PHx, which was associated with increased serum TNF and IL-6 levels [51]. Therefore, it has become clear that the MCP-1 produced by CD68^+^ Kupffer cells after GdCl_3_ treatment induces the accumulation of CD11b^+^ Kupffer cells/Mφ and increases their TNF and IL-6 production [49], thus resulting in increased hepatocyte proliferation after PHx.

Although further studies are required to elucidate the precise role of CD68^+^ Kupffer cells in various immune phenomena, including hepatocyte regeneration, it should be kept in mind that the depletion of CD68^+^ Kupffer cells made mice extremely susceptible to bacterial infection [23]. On the other hand, the TNF produced by CD11b^+^ Kupffer cells/Mφ is required for the activation and ROS production of CD68^+^ Kupffer cells [44], suggesting that CD11b^+^ Kupffer cells/Mφ and their TNF production is required for the bactericidal activity of CD68^+^ Kupffer cells. Tools to temporarily deplete either Kupffer cell subset, namely non-lethal irradiation for depleting CD11b^+^ Kupffer cells and clodronate-liposomes for depleting CD68^+^ Kupffer cells [23, 24], may facilitate the delineation of the characteristics of both Kupffer cell subsets. In addition, we have recently observed that non-lethal irradiation (5 Gy) does not affect the phagocytic and bactericidal activity of CD68^+^ Kupffer cells (our unpublished observation), thus suggesting that the function of CD68^+^ Kupffer cells is retained after irradiation. Taken together, the concept of two liver Kupffer cell subsets may provide new insight into understanding their interactions/cooperation with each other and with other liver lymphocytes under various physiological and pathological conditions in the liver.

In conclusion, following NKT cell activation during the early phase after PHx, CD11b^+^ Kupffer cells/Mφ increase, are activated and produce TNF and FasL, which play a pivotal role in hepatocyte regeneration during the middle stage of liver regeneration, and NK cells may terminate hepatocyte regeneration [7, 21].

**Fig 7:**
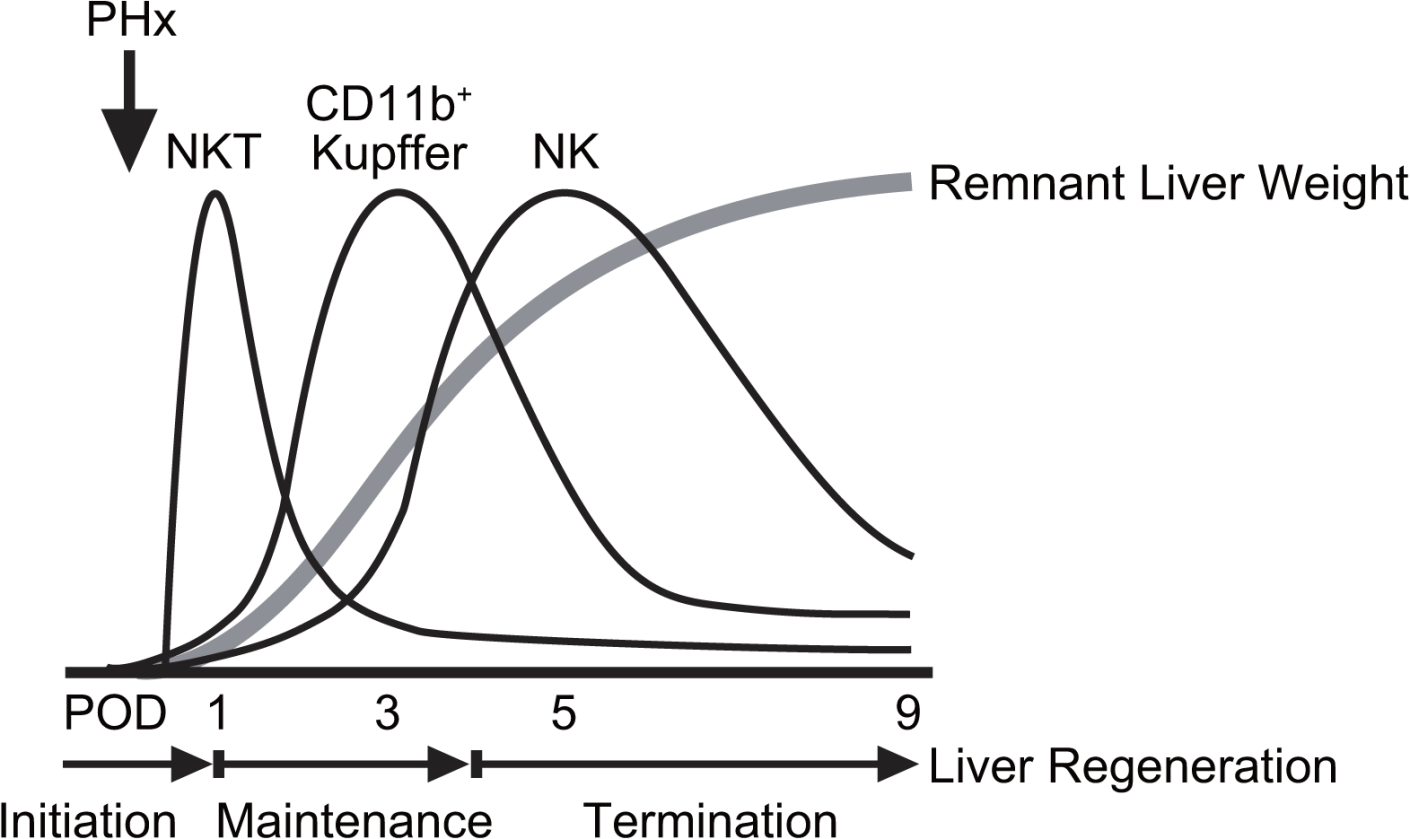
The schematic diagram of the time course of the remnant liver weight recovery after PHx and the liver immune cells in each stage of liver regeneration.

Since NK cells were reported to be cytotoxic against regenerating hepatocytes [21], a schematic diagram of the time course of liver regeneration, liver immune cells and their function after PHx is shown (Fig 7).

## Supporting Information

S1 ARRIVE Checklist ARRIVE Checklist. (PDF)

S1 Fig Intracellular TNF content of liver MNCs in Sham operated mice. (EPS)

S2 Fig The flow cytometric analysis of liver F4/80^+^CD11b^+^ cells and F4/80^+^CD68^+^ cells in control or 5 Gy irradiated PHx mice. 5 days after irradiation, mice were subjected to PHx, and in POD 3, liver MNCs were isolated with collagenase digestion. F4/80 positive population was gated and dot plot analysis was performed with CD11b and CD68.
(EPS)

S3 Fig Remnant liver NK and NKT cell population of 5 Gy irradiated sham or PHx mice. 5 days after irradiation, PHx or sham operation was performed. Remnant liver was removed 12 hours after operation, and MNCs were isolated without collagenase digestion.
(EPS)

S4 Fig Cytotoxic assay of remnant liver MNCs against EL-4 or Yac-1. Liver MNCs were isolated 3, 7, or 14 days after PHx or sham operation and incubated with ^51^Cr incorporated EL-4 or Yac-1 cells. Supernatant ^51^Cr release was evaluated with gamma counting device.
(EPS)

S5 Fig Mice survival after EL-4 cell inoculation. 3 days or 7 days after PHx or sham operation, mice were injected with EL-4 cells (1×10^6^) via tail vein. Mice survival rates were observed.
(EPS)
